# An efficient synthesis of novel sucrose-containing dilactams

**DOI:** 10.1007/s00706-012-0894-2

**Published:** 2013-01-17

**Authors:** Mykhaylo A. Potopnyk, Sławomir Jarosz

**Affiliations:** Institute of Organic Chemistry, Polish Academy of Sciences, Kasprzaka 44/52, 01-224 Warsaw, Poland

**Keywords:** Carbohydrates, Macrocycles, Alkylation, Reductions, Cyclization

## Abstract

**Abstract:**

An efficient and convenient approach to sucrose-containing dilactams has been developed. The method, based on reaction of regioisomeric 6,6′-di-*O*-[(aminomethyl)-phenyl]-1′,2,3,3′,4,4′-hexa-*O*-methylsucrose with isophtaloyl or 2,6-pyridinedicarbonyl dichlorides, provided the 1:1-macrocycles in good yields.

**Graphical Abstract:**

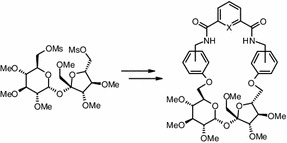
.

## Introduction

Macrocyclic compounds are important in supramolecular chemistry [1]. Especially interesting are chiral receptors capable of enantioselective complexation of a variety of important chiral guests. Carbohydrates, inexpensive, renewable raw materials available optically pure, are particularly useful in planning and executing the synthesis of such chiral hosts. The different configurations and conformations of carbohydrates can be incorporated in the target macrocycle, which makes these compounds convenient chiral synthetic analogs of poly(ethylene glycol) (PEG) reagents [2].

Chiral crown and aza-crown ethers with carbohydrate scaffolds have been extensively used as chiral catalysts in asymmetric synthesis (asymmetric epoxidation of chalcones [3–5], Michael addition [3, 4, 6, 7], and Darzens reactions [3–5, 7, 8]). Carbohydrate-containing macrocycles have also been investigated as fluorescent molecular sensors for cations [9, 10] and anions [11].

Sucrose, the most common disaccharide occurring in nature, is a promising building block for synthesis of such chiral macrocyclic receptors [12–14]. Its aza-crown derivatives enabled highly enantioselective complexation of the (*S*)-1-phenylethylammonium cation [15].

Isophthalic and pyridine-2,6-diamides, because of their proton-donor properties, are convenient scaffolds used as building blocks in the synthesis of macrocyclic receptors designed for complexation of anions [16], ion pairs [17], zwitterions [18], and amino acid derivatives [19]. The anion-complexing properties of such diamides have been exploited in templated syntheses of catenane [20] and rotaxane [21] systems. Macrocycles incorporating the pyridine-2,6-diamide functionality are known as molecular turnstiles [22]. Combination of the sucrose scaffold with isophthalic or pyridine-2,6-diamide units may be useful means of synthesis of a new type of chiral receptor with interesting properties.

Very recently, we reported an effective procedure for synthesis of 1′,2,3,3′,4,4′-hexa-*O*-methyl-6,6′-di-*O*-(methylsulfonyl)sucrose (**1**; four steps, 48 % overall yield) which was used for preparation of macrocyclic bis-amides **3a**–**3c** and **4a**–**4c** (Scheme 1). Condensation of dimesylate **1** with 2 equiv. of the appropriate nitrophenol (*ortho*, *meta*, or *para*) followed by reduction of the nitro groups provided the expected 6,6′-di-*O*-(aminophenyl)-1′,2,3,3′,4,4′-hexa-*O*-methylsucroses (**2a**–**2c**). Reaction of dianilines **2a** and **2b** (*o* or *m*) with isophthaloyl or 2,6-pyridinedicarbonyl dichlorides (**5** and **6**) afforded the monomeric macrocycles in excellent yields, whereas reaction of the *p*-diamines furnished dimers as the major products (Scheme 1), with smaller amounts of the expected monomers **3c**, **4c** [23].

**Figure:**
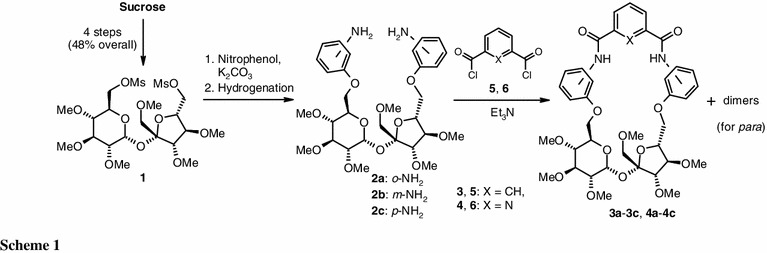

A crucial aspect of the synthesis of this type of receptor is the relative orientation of the two amino groups in the energetically accessible conformations of the substrates. The amino groups in the *p*-substituted derivative **2c** are rather distant from each other (compared with the *o* and *m* analogs **2a** and **2b**). Thus, the intermediate formed in reaction of the acid dichloride (**5** or **6**) with the first amino group will react preferentially with a second molecule of **2c** (to form the dimer) rather than undergo the intramolecular process leading to **3c** or **4c** [23].

## Results and Discussion

In this paper we report the synthesis of new sucrose macrocyclic derivatives which are twice homologated compared with compounds **3a**–**3c** and **4a**–**4c**. Arylmethaneamines **9a**–**9c** (the homologated analogs of anilines **2a**–**2c**) were used as starting materials for the preparation of conformationally less demanding structures.

**Fig. 1:**
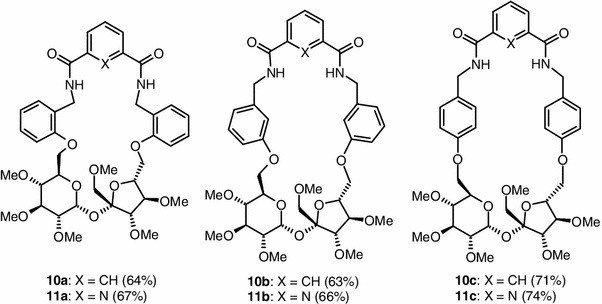
Macrocyclic diamides **10a**–**10c** and **11a**–**11c**

1′,2,3,3′,4,4′-Hexa-*O*-methyl-6,6′-di-*O*-(methylsulfonyl)sucrose (**1**) was treated with 2 equiv. of the appropriate, commercially available cyanophenol (**7a**–**7c**; *o*, *m*, *p*, respectively) in DMF in the presence of potassium carbonate to give the corresponding 6,6′-di-*O*-(cyanophenyl)-1′,2,3,3′,4,4′-hexa-*O*-methylsucroses (**8a**–**8c**) in 81–84 % yield. These compounds were quantitatively converted into the 6,6′-di-*O*-[(aminomethyl)phenyl]-1′,2,3,3′,4,4′-hexa-*O*-methylsucroses (**9a**–**9c**) by reduction with LiAlH_4_. The crude bis-amines **9a**–**9c** were subjected to cyclocondensation reaction with isophthaloyl or 2,6-pyridinedicarbonyl dichlorides (**5** and **6**, respectively) to achieve closure of the ring (Scheme 2). To avoid formation of the dimeric byproducts, the reactions were performed in dilute solution. In all cases a 1:1-product (**10a**–**10c** and **11a**–**11c**) was formed in good yield (63–74 %; Fig. 1).

**Figure:**
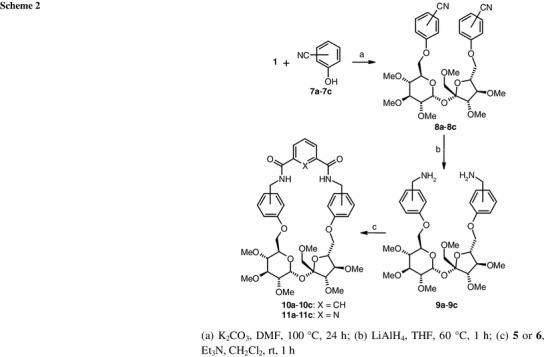

In summary, we have developed a simple, rapid, and efficient procedure for preparation of sucrose-based promising optically active receptors. Because of the conformational mobility (less rigid structure) of the diamine **9c**, which differ from **2c** (which furnishes both the monomers and the dimers in the reaction with dichlorides **5** or **6**; Scheme 1) only in the length of the chain, we were able to suppress formation of the dimer and obtain monomeric macrocycles in good yield. This strategy was applicable to the synthesis of sucrose-derived macrocycles containing isophthalic and pyridine-2,6-diamide groups.

## Experimental

All reported NMR spectra were recorded with a Varian Vnmrs-600 MHz spectrometer (at 600 and 150 MHz for ^1^H and ^13^C NMR spectra, respectively); solutions were prepared in CDCl_3_ with TMS as the internal standard. Most of the resonances were assigned by COSY (^1^H–^1^H) and gradient selected HSQC and HMBC correlations. IR spectra (CHCl_3_, film) were recorded on a Perkin Elmer FT-IR Spectrum 2000. Mass spectra were recorded with an ESI/MS Mariner (PerSeptive Biosystem) mass spectrometer. Elemental analysis was performed with a Perkin-Elmer 2400 CHN analyzer; results agreed satisfactorily with calculated values. Optical rotation was measured with a Jasco DIP-360 digital polarimeter; solutions were prepared in CH_2_Cl_2_ (*c* = 1). Flash chromatography was performed on silica gel (Merck, 230–400 mesh). The organic phases were dried over anhydrous magnesium sulfate.

### *General procedure for preparation of 6,6′*-*di*-*O*-*(cyanophenyl)*-*1′,2,3,3′,4,4′*-*hexa*-*O*-*methylsucroses (****8a***–***8c****)*

To a solution of 291 mg compound **1** (0.5 mmol) in 25 cm^3^ dry DMF, 345 mg K_2_CO_3_ (2.5 mmol) was added than 179 mg of the corresponding cyanophenol **7a**–**7c** (1.5 mmol). The mixture was stirred for 24 h at 100 °C then cooled to room temperature. Water (50 cm^3^) and 50 cm^3^ AcOEt were added, the organic phase was separated, and the aqueous phase was extracted with ethyl acetate (4 × 50 cm^3^). Combined organic solutions were washed with water (2 × 30 cm^3^), 30 cm^3^ brine, dried, concentrated, and the product was isolated by flash chromatography (hexane–ethyl acetate, 9:1 to 7:3) to afford **8a**–**8c**.

#### *6,6′*-*Di*-*O*-*(2*-*cyanophenyl)*-*1′,2,3,3′,4,4′*-*hexa*-*O*-*methylsucrose* (**8a**, C_32_H_40_N_2_O_11_)

Colorless oil; yield: 255 mg (81 %); TLC: *R*_*f*_ = 0.47 (hexane/AcOEt 1:2); [*α*]_D_^24^ = +55.9° cm^2^ g^−1^; IR: $\overline{v}$ = 2,983, 2,934, 2,832, 2,228, 1,741, 1,599, 1,581, 1,494, 1,449, 1,374, 1,292, 1,261, 1,185, 1,164, 1,102, 1,045, 1,018, 983, 879, 835, 757, 667, 566, 497 cm^−1^; ^1^H NMR: *δ* = 7.55 (dd, *J* = 7.7 Hz, 1.7 Hz, 1H, Ar), 7.53 (dd, *J* = 7.5 Hz, 1.7 Hz, 1H, Ar), 7.51 (ddd, *J* = 8.4 Hz, 7.6 Hz, 1.6 Hz, 1H, Ar), 7.37 (ddd, *J* = 8.5 Hz, 7.6 Hz, 1.7 Hz, 1H, Ar), 6.99–7.07 (m, 3H, Ar), 6.95 (dd, *J* = 7.6 Hz, 7.6 Hz, 1H, Ar), 5.59 (d, *J*_1,2_ = 3.7 Hz, 1H, H-1), 4.32–4.38 (m, 2H, 2H-6′), 4.23–4.27 (m, 3H, H-5′, 2H-6), 4.18 (m, 1H, H-5), 4.09 (d, *J*_3′,4′_ = 6.7 Hz, 1H, H-3′), 3.90 (dd, *J*_4′,3′_ = 6.7 Hz, *J*_4′,5′_ = 6.1 Hz, 1H, H-4′), 3.66 (d, *J*_1′,1′_ = 11.1 Hz, 1H, H-1′), 3.60 (s, 3H, CH_3_), 3.54 (s, 3H, CH_3_), 3.51 (dd, *J*_3,2_ = 9.7 Hz, *J*_3,4_ = 9.1 Hz, 1H, H-3), 3.49 (s, 3H, CH_3_), 3.47 (s, 3H, CH_3_), 3.46 (s, 3H, CH_3_), 3.43 (d, *J*_1′,1′_ = 11.1 Hz, 1H, H-1′), 3.42 (s, 3H, CH_3_), 3.34 (dd, *J*_4,3_ = 9.1 Hz, *J*_4,5_ = 10.0 Hz, 1H, H-4), 3.16 (dd, *J*_2,1_ = 3.7 Hz, *J*_2,3_ = 9.7 Hz, 1H, H-2) ppm; ^13^C NMR: *δ* = 160.49, 160.33, 134.33, 134.15, 133.82, 133.82, 121.13, 121.01, 116.40, 116.30, 112.87, 112.79 (12 × C–Ar), 104.98 (C-2′), 102.33 (CN), 102.25 (CN), 90.02 (C-1), 85.88 (C-3′), 84.92 (C-4′), 83.12 (C-3), 81.46 (C-2), 79.16 (C-4), 78.83 (C-5′), 73.30 (C-1′), 70.42 (C-6′), 69.71 (C-5), 68.52 (C-6), 60.63, 60.47, 59.51, 58.75, 58.55, 58.46 (6 × OCH_3_) ppm; HRMS (ESI): calcd for C_32_H_40_N_2_O_11_Na [M + Na]^+^ 651.2524, found 651.2525.

#### *6,6′*-*Di*-*O*-*(3*-*cyanophenyl)*-*1′,2,3,3′,4,4′*-*hexa*-*O*-*methylsucrose* (**8b**, C_32_H_40_N_2_O_11_)

Colorless oil; yield: 265 mg (84 %); TLC: *R*_*f*_ = 0.51 (hexane/AcOEt 1:2); [*α*]_D_^22^ = +56.3° cm^2^ g^−1^; IR: $\overline{v}$ = 3,075, 2,982, 2,933, 2,831, 2,231, 1,741, 1,597, 1,579, 1,483, 1,432, 1,328, 1,291, 1,265, 1,185, 1,148, 1,101, 1,017, 983, 873, 790, 756, 682, 616, 517, 475 cm^−1^; ^1^H NMR: *δ* = 7.36 (dd, *J* = 7.8 Hz, 8.0 Hz, 1H, Ar), 7.28 (dd, *J* = 7.8 Hz, 8.2 Hz, 1H, Ar), 7.24 (d, *J* = 7.6 Hz, 1H, Ar), 7.20 (d, *J* = 7.4 Hz, 1H, Ar), 7.12–7.17 (m, 3H, Ar), 7.11 (dd, *J* = 8.2 Hz, 2.3 Hz, 1H, Ar), 5.60 (d, *J*_1,2_ = 3.7 Hz, 1H, H-1), 4.29 (m, 1H, H-6′), 4.12–4.23 (m, 5H, H-5, H-5′, 2H-6, H-6′), 4.11 (d, *J*_3′,4′_ = 7.6 Hz, 1H, H-3′), 3.97 (dd, *J*_4′,3′_ = 7.6 Hz, *J*_4′,5′_ = 7.3 Hz, 1H, H-4′), 3.64 (s, 3H, CH_3_), 3.61 (d, *J*_1′,1′_ = 11.0 Hz, 1H, H-1′), 3.53 (s, 3H, CH_3_), 3.51 (s, 3H, CH_3_), 3.49 (m, 1H, H-3), 3.47 (s, 3H, CH_3_), 3.44 (s, 3H, CH_3_), 3.43 (s, 3H, CH_3_), 3.43 (d, *J*_1′,1′_ = 11.0 Hz, 1H, H-1′), 3.22 (dd, *J* = 10.2 Hz, 8.9 Hz, 1H, H-4), 3.16 (dd, *J*_2,1_ = 3.7 Hz, *J*_2,3_ = 9.7 Hz, 1H, H-2) ppm; ^13^C NMR: *δ* = 158.74, 158.65, 130.47, 130.26, 124.83, 124.76, 119.90, 119.84, 118.51, 118.51, 117.48, 117.39 (12 × C–Ar), 113.30 (CN), 113.16 (CN), 104.39 (C-2′), 89.39 (C-1), 85.34 (C-3′), 83.79 (C-4′), 83.21 (C-3), 81.64 (C-2), 79.47 (C-4), 78.53 (C-5′), 73.70 (C-1′), 69.66 (C-5), 69.30 (C-6′), 67.80 (C-6), 60.76, 60.58, 59.42, 58.65, 58.47, 58.43 (6 × OCH_3_) ppm; HRMS (ESI): calcd for C_32_H_40_N_2_O_11_Na [M + Na]^+^ 651.2524, found 651.2522.

#### *6,6′*-*Di*-*O*-*(4*-*cyanophenyl)*-*1′,2,3,3′,4,4′*-*hexa*-*O*-*methylsucrose* (**8c**, C_32_H_40_N_2_O_11_)

Colorless oil; yield: 258 mg (82 %); TLC: *R*_*f*_ = 0.54 (hexane/AcOEt 1:2); [*α*]_D_^24^ = +75.8° cm^2^ g^−1^; IR: $\overline{v}$ = 2,983, 2,933, 2,831, 2,225, 1,606, 1,575, 1,509, 1,453, 1,419, 1,374, 1,302, 1,259, 1,173, 1,150, 1,100, 1,019, 983, 836, 755, 724, 684, 548 cm^−1^; ^1^H NMR: *δ* = 7.59 (d, *J* = 9.0 Hz, 2H, Ar), 7.50 (d, *J* = 9.0 Hz, 2H, Ar), 6.98 (d, *J* = 9.0 Hz, 2H, Ar), 6.94 (d, *J* = 9.0 Hz, 2H, Ar), 5.60 (d, *J*_1,2_ = 3.7 Hz, 1H, H-1), 4.32 (m, 1H, H-6′), 4.14–4.22 (m, 5H, H-5, 2H-6, H-5′, H-6′), 4.10 (d, *J*_3′,4′_ = 7.5 Hz, 1H, H-3′), 3.93 (dd, *J*_4′,3′_ = 7.5 Hz, *J*_4′,5′_ = 7.3 Hz, 1H, H-4′), 3.62 (s, 3H, CH_3_), 3.61 (d, *J*_1′,1′_ = 10.8 Hz, 1H, H-1′), 3.52 (s, 3H, CH_3_), 3.49 (s, 3H, CH_3_), 3.49 (dd, *J*_3,2_ = 9.7 Hz, *J*_3,4_ = 8.9 Hz, 1H, H-3), 3.435 (s, 3H, CH_3_), 3.432 (s, 3H, CH_3_), 3.427 (d, *J*_1′,1′_ = 10.8 Hz, 1H, H-1′), 3.418 (s, 3H, CH_3_), 3.20 (dd, *J*_4,3_ = 8.9 Hz, *J*_4,5_ = 9.7 Hz, 1H, H-4), 3.15 (dd, *J*_2,1_ = 3.7 Hz, *J*_2,3_ = 9.7 Hz, 1H, H-2) ppm; ^13^C NMR: *δ* = 161.92, 161.89, 134.11, 133.93, 119.03, 118.94, 115.33, 115.27 (12 × C–Ar), 104.54 (CN), 104.50 (C-2′), 104.36 (CN), 89.46 (C-1), 85.35 (C-3′), 83.64 (C-4′), 83.24 (C-3), 81.70 (C-2), 79.47 (C-4), 78.49 (C-5′), 73.68 (C-1′), 69.51 (C-5), 69.33 (C-6′), 67.78 (C-6), 60.78, 60.63, 59.46, 58.72, 58.51, 58.39 (6 × OCH_3_) ppm; HRMS (ESI): calcd for C_32_H_40_N_2_O_11_Na [M + Na]^+^ 651.2524, found 651.2538.

### *General procedure for synthesis of 6,6′*-*di*-*O*-*[4*-*(aminomethyl)phenyl]*-*1′,2,3,3′,4,4′*-*hexa*-*O*-*methylsucroses (****9a***–***9c****)*

To a cooled (0 °C) solution of 215 mg compound **8a**–**8c** (0.34 mmol) in 30 cm^3^ dry THF, 93 mg LiAlH_4_ (2.45 mmol) was added slowly within 5 min. The mixture was stirred for 1 h at 60 °C and cooled to room temperature. Excess hydride was carefully decomposed with 10 cm^3^ water and 40 cm^3^ aqueous potassium bisulfate (KHSO_4_). Ethyl acetate (50 cm^3^) was added, the layers were separated, and the aqueous layer was extracted with ethyl acetate (3 × 40 cm^3^). The combined organic solutions were dried, concentrated, and the crude product was used in the next step without purification.

### *General procedure for syntheses of macrocyclic diamides****10a***–***10c****and****11a***–***11c***

This reaction was conducted under an argon atmosphere: 35 mg isophthaloyl or 2,6-pyridinedicarbonyl dichloride (**5** or **6**, 0.17 mmol) was dissolved in 20 cm^3^ dry CH_2_Cl_2_ and added dropwise to a stirred solution of 108 mg diamine **9a**–**9c** (0.17 mmol) in 40 cm^3^ dry CH_2_Cl_2_ containing 71 mm^3^ Et_3_N (0.51 mmol), and the mixture was stirred for 1 h at room temperature. The resulting solution was concentrated in vacuum and the residue was dissolved in 40 cm^3^ ethyl acetate and 20 cm^3^ water. Saturated K_2_CO_3_ solution (10 cm^3^) was added, the layers were separated, and the aqueous layer was extracted with ethyl acetate (3 × 30 cm^3^). The combined organic extracts were washed with 20 cm^3^ water and 10 cm^3^ brine, dried, concentrated, and the products were isolated by flash chromatography (hexane–ethyl acetate, 50:50 to 25:75).

#### *6,6′*-*Di*-*O*-*[[benzene*-*1,3*-*diyl*-*bis(carbonylaminomethyl)]*-*2,2′*-*diphenyl]*-*1′,2,3,3′,4,4′*-*hexa*-*O*-*methylsucrose* (**10a**, C_40_H_50_N_2_O_13_)

White solid; yield: 84 mg (64 %); m.p.: 134 °C; TLC: *R*_*f*_ = 0.35 (AcOEt); [*α*]_D_^22^ = +78.8° cm^2^ g^−1^; IR: $\overline{v}$ = 3,347, 3,064, 2,982, 2,933, 2,830, 1,658, 1,603, 1,590, 1,526, 1,495, 1,451, 1,359, 1,318, 1,293, 1,250, 1,186, 1,161, 1,100, 1,049, 1,017, 1,004, 982, 941, 882, 825, 753, 710, 593, 527 cm^−1^; ^1^H NMR: *δ* = 8.01–8.05 (m, 2H, isophthalic), 7.57 (s, 1H, isophthalic), 7.52 (t, *J* = 7.7 Hz, 1H, isophthalic), 7.32 (d, *J* = 7.3 Hz, 1H, Ar), 7.26–7.31 (m, 3H, Ar), 6.92–6.96 (m, 2H, Ar), 6.90 (d, *J* = 8.5 Hz, 1H, Ar), 6.85 (br s, 1H, NH), 6.74 (d, *J* = 8.0 Hz, 1H, Ar), 6.66 (br s, 1H, NH), 4.72–4.77 (m, 2H, CH_2_N), 4.59 (d, *J*_1,2_ = 3.3 Hz, 1H, H-1), 4.51 (dd, *J* = 13.8 Hz, 6.2 Hz, 1H, CH_2_N), 4.45 (dd, *J* = 13.6 Hz, 6.5 Hz, 1H, CH_2_N), 4.27 (dd, *J*_6′,6′_ = 9.9 Hz, *J*_6′,5′_ = 2.3 Hz, 1H, H-6′), 4.14–4.20 (m, 2H, H-5′, H-6), 4.08 (d, *J*_3′,4′_ = 7.4 Hz, 1H, H-3′), 3.93–3.97 (m, 2H, H-5, H-6′), 3.76 (dd, *J*_6,6_ = 10.0 Hz, *J*_6,5_ = 1.5 Hz, 1H, H-6), 3.70 (dd, *J*_4′,5′_ = 7.5 Hz, *J*_4′,3′_ = 7.4 Hz, 1H, H-4′), 3.56 (s, 3H, CH_3_), 3.47 (s, 3H, CH_3_), 3.46 (m, 1H, H-3), 3.440 (s, 3H, CH_3_), 3.435 (s, 3H, CH_3_), 3.40–3.43 (m, 2H, H-1′, H-4), 3.39 (s, 3H, CH_3_), 3.23 (s, 3H, CH_3_), 3.14 (d, *J*_1′,1′_ = 11.2 Hz, 1H, H-1′), 2.75 (dd, *J*_2,1_ = 3.3 Hz, *J*_2,3_ = 9.5 Hz, 1H, H-2) ppm; ^13^C NMR: *δ* = 166.96 (C=O), 166.75 (C=O), 156.85, 156.81 (2 × C–Ar), 135.53, 135.42, 131.21, 130.87 (4 × C-isophthalic), 131.09, 130.84, 129.43, 129.31 (4 × C–Ar), 129.16 (C-isophthalic), 127.05, 125.73 (2 × C–Ar), 123.80 (C-isophthalic), 121.72, 121.10, 112.51, 110.99 (4 × C–Ar), 104.37 (C-2′), 90.41 (C-1), 84.70 (C-3′), 84.09 (C-4′), 82.72 (C-3), 81.31 (C-2), 78.59 (C-4), 77.87 (C-5′), 73.53 (C-1′), 70.95 (C-6′), 70.25 (C-5), 66.07 (C-6), 60.67, 60.33, 59.67, 58.87, 58.04, 57.99 (6 × OCH_3_), 41.53, 40.94 (2 × CH_2_N) ppm; HRMS (ESI): calcd for C_40_H_50_N_2_O_13_Na [M + Na]^+^ 789.3205, found 789.3228.

#### *6,6′*-*Di*-*O*-*[[pyridine*-*1,3*-*diyl*-*bis(carbonylaminomethyl)]*-*2,2′*-*diphenyl]*-*1′,2,3,3′,4,4′*-*hexa*-*O*-*methylsucrose* (**11a**, C_39_H_49_N_3_O_13_)

White solid; yield: 88 mg (67 %); m.p.: 96 °C; TLC: *R*_*f*_ = 0.37 (AcOEt); [*α*]_D_^22^ = +163.1° cm^2^ g^−1^; IR: $\overline{v}$ = 3,537, 3,403, 3,303, 3,064, 2,984, 2,933, 2,831, 1,735, 1,674, 1,602, 1,590, 1,528, 1,494, 1,452, 1,360, 1,289, 1,278, 1,244, 1,186, 1,161, 1,149, 1,101, 1,051, 1,017, 1,003, 983, 945, 878, 844, 754, 683, 647, 609, 564 cm^−1^; ^1^H NMR: *δ* = 8.48 (dd, *J* = 4.3 Hz, 7.3 Hz, 1H, NH), 8.34–8.37 (m, 2H, pyridine), 8.22 (dd, *J* = 4.1 Hz, 7.6 Hz, 1H, NH), 8.02 (t, *J* = 7.8 Hz, 1H, pyridine), 7.39 (dd, *J* = 7.5 Hz, 1.6 Hz, 1H, Ar), 7.30 (dd, *J* = 7.6 Hz, 1.6 Hz, 1H, Ar), 7.23–7.29 (m, 2H, Ar), 6.99 (dd, *J* = 8.2 Hz, 0.8 Hz, 1H, Ar), 6.93 (ddd, *J* = 7.5 Hz, 7.4 Hz, 0.9 Hz, 1H, Ar), 6.88 (ddd, *J* = 7.6 Hz, 7.4 Hz, 0.8 Hz, 1H, Ar), 6.99 (dd, *J* = 8.2 Hz, 0.9 Hz, 1H, Ar), 5.35 (d, *J*_1,2_ = 3.4 Hz, 1H, H-1), 4.85 (dd, *J* = 14.4 Hz, 4.3 Hz, 1H, CH_2_N), 4.78 (dd, *J* = 14.2 Hz, 4.1 Hz, 1H, CH_2_N), 4.69 (dd, *J* = 14.4 Hz, 7.3 Hz, 1H, CH_2_N), 4.61 (dd, *J* = 14.2 Hz, 7.6 Hz, 1H, CH_2_N), 4.34 (dd, *J*_6′,6′_ = 10.2 Hz, *J*_6′,5′_ = 3.1 Hz, 1H, H-6′), 4.22 (m, 1H, H-5′), 4.11 (d, *J*_3′,4′_ = 6.9 Hz, 1H, H-3′), 4.01–4.07 (m, 2H, H-5, H-6), 3.98 (dd, *J*_6′,5′_ = 7.7 Hz, *J*_6′,6′_ = 10.2 Hz, 1H, H-6′), 3.86 (dd, *J*_4′,3′_ = 6.9 Hz, *J*_4′,5′_ = 6.9 Hz, 1H, H-4′), 3.76 (d, *J*_6,6_ = 9.2 Hz, 1H, H-6), 3.57 (d, *J*_1′,1′_ = 11.0 Hz, 1H, H-1′), 3.52 (s, 3H, CH_3_), 3.48 (dd, *J*_3,2_ = 9.6 Hz, *J*_3,4_ = 9.0 Hz, 1H, H-3), 3.460 (s, 3H, CH_3_), 3.457 (s, 3H, CH_3_), 3.450 (s, 3H, CH_3_), 3.42 (s, 3H, CH_3_), 3.41 (d, *J*_1′,1′_ = 11.0 Hz, 1H, H-1′), 3.32 (dd, *J*_4,3_ = 9.0 Hz, *J*_4,5_ = 9.6 Hz, 1H, H-4), 3.25 (s, 3H, CH_3_), 2.81 (dd, *J*_2,1_ = 3.4 Hz, *J*_2,3_ = 9.6 Hz, 1H, H-2) ppm; ^13^C NMR: *δ* = 163.38 (C=O), 163.12 (C=O), 156.67, 156.64 (2 × C–Ar), 148.98, 148.89, 138.83 (3 × C-pyridine), 130.72, 130.02, 129.03, 128.71, 128.34, 126.42 (6 × C–Ar), 124.74, 124.74 (2 × C-pyridine), 122.67, 121.43, 114.94, 112.44 (4 × C–Ar), 104.92 (C-2′), 89.89 (C-1), 84.85 (C-3′), 84.19 (C-4′), 82.81 (C-3), 81.88 (C-2), 79.03 (C-4), 78.69 (C-5′), 73.65 (C-1′), 72.06 (C-6′), 70.26 (C-5), 67.30 (C-6), 60.48, 60.42, 59.54, 58.50, 58.38, 58.03 (6 × OCH_3_), 39.65, 38.14 (2 × CH_2_N) ppm; HRMS (ESI): calcd for C_39_H_49_N_3_O_13_Na [M + Na]^+^ 790.3158, found 790.3165.

#### *6,6′*-*Di*-*O*-*[[benzene*-*1,3*-*diyl*-*bis(carbonylaminomethyl)]*-*3,3′*-*diphenyl]*-*1′,2,3,3′,4,4′*-*hexa*-*O*-*methylsucrose* (**10b**, C_40_H_50_N_2_O_13_)

White solid; yield: 82 mg (63 %); m.p.: 111 °C; TLC: *R*_*f*_ = 0.36 (AcOEt); [*α*]_D_^22^ = +59.8° cm^2^ g^−1^; IR: $\overline{v}$ = 3,333, 2,981, 2,931, 2,830, 1,654, 1,599, 1,586, 1,535, 1,487, 1,448, 1,358, 1,290, 1,267, 1,237, 1,183, 1,151, 1,100, 1,056, 1,017, 997, 983, 956, 876, 755, 691, 622 cm^−1^; ^1^H NMR: *δ* = 7.97–8.01 (m, 2H, isophthalic), 7.91 (s, 1H, isophthalic), 7.52 (dd, *J* = 7.8 Hz, 7.8 Hz, 1H, isophthalic), 7.24 (dd, *J* = 7.8 Hz, 7.9 Hz, 1H, Ar), 7.19 (dd, *J* = 7.8 Hz, 8.0 Hz, 1H, Ar), 6.97 (s, 1H, Ar), 6.91 (d, *J* = 7.8 Hz, 1H, Ar), 6.87–6.89 (m, 2H, Ar), 6.82–6.86 (m, 2H, Ar), 6.69 (dd, *J* = 5.6 Hz, 5.6 Hz, 1H, NH), 6.64 (dd, *J* = 5.6 Hz, 5.6 Hz, 1H, NH), 5.70 (d, *J*_1,2_ = 3.8 Hz, 1H, H-1), 4.65 (m, 2H, CH_2_N), 4.50 (dd, *J* = 14.7 Hz, 5.6 Hz, 1H, CH_2_N), 4.42 (dd, *J* = 14.5 Hz, 5.6 Hz, 1H, CH_2_N), 4.12–4.23 (m, 4H, H-5, H-6, 2 × H-6′), 4.06–4.12 (m, 1H, H-5′), 4.09 (d, *J*_3′,4′_ = 8.0 Hz, 1H, H-3′), 4.03 (br d, *J*_6,6_ = 9.1 Hz, 1H, H-6), 3.97 (dd, *J*_4′,3′_ = 8.0 Hz, *J*_4′,5′_ = 8.0 Hz, 1H, H-4′), 3.61 (s, 3H, CH_3_), 3.58 (d, *J*_1′,1′_ = 10.9 Hz, 1H, H-1′), 3.49 (s, 3H, CH_3_), 3.48 (dd, *J*_3,2_ = 9.6 Hz, *J*_3,4_ = 9.3 Hz, 1H, H-3), 3.45 (s, 3H, CH_3_), 3.42 (d, *J*_1′,1′_ = 10.9 Hz, 1H, H-1′), 3.41 (s, 6H, 2CH_3_), 3.40 (s, 3H, CH_3_), 3.36 (dd, *J*_4,3_ = 9.3 Hz, *J*_4,5_ = 9.6 Hz, 1H, H-4), 3.21 (dd, *J*_2,1_ = 3.8 Hz, *J*_2,3_ = 9.6 Hz, 1H, H-2) ppm; ^13^C NMR: *δ* = 166.71 (C=O), 166.34 (C=O), 159.20, 159.01, 139.63, 139.28 (4 × C–Ar), 134.69, 134.56 (2 × C-isophthalic), 130.78 (2C-isophthalic), 129.79, 129.77 (2 × C–Ar), 129.39, 124.05 (2 × C-isophthalic), 121.64, 120.80, 115.13, 114.48, 113.75, 112.49 (6 × C–Ar), 104.15 (C-2′), 88.69 (C-1), 85.02 (C-3′), 83.17 (C-3), 83.03 (C-4′), 81.29 (C-2), 79.14 (C-4), 78.08 (C-5′), 74.83 (C-1′), 69.66 (C-5), 68.95 (C-6′), 66.30 (C-6), 60.65, 60.46, 59.41, 58.59, 58.39, 58.03 (6 × OCH_3_), 44.25, 43.95 (2 × CH_2_N) ppm; HRMS (ESI): calcd for C_40_H_50_N_2_O_13_Na [M + Na]^+^ 789.3202, found 789.3214.

#### *6,6′*-*Di*-*O*-*[[pyridine*-*1,3*-*diyl*-*bis(carbonylaminomethyl)]*-*3,3′*-*diphenyl]*-*1′,2,3,3′,4,4′*-*hexa*-*O*-*methylsucrose* (**11b**, C_39_H_49_N_3_O_13_)

White solid; yield: 86 mg (66 %); m.p.: 125 °C; TLC: *R*_*f*_ = 0.39 (AcOEt); [*α*]_D_^22^ = +61.6° cm^2^ g^−1^; IR: $\overline{v}$ = 3,317, 2,980, 2,930, 2,831, 1,679, 1,661, 1,599, 1,586, 1,532, 1,488, 1,448, 1,358, 1,312, 1,287, 1,271, 1,237, 1,180, 1,148, 1,101, 1,057, 1,038, 1,019, 1,002, 982, 876, 844, 755, 682, 647, 623 cm^−1^; ^1^H NMR: *δ* = 8.34–8.38 (m, 2H, pyridine), 8.04 (t, *J* = 7.8 Hz, 1H, pyridine), 7.90 (dd, *J* = 5.1 Hz, 6.6 Hz, 1H, NH), 7.85 (dd, *J* = 5.6 Hz, 5.6 Hz, 1H, NH), 7.25 (dd, *J* = 7.5 Hz, 7.9 Hz, 1H, Ar), 7.10 (dd, *J* = 7.9 Hz, 7.9 Hz, 1H, Ar), 6.92 (d, *J* = 7.5 Hz, 1H, Ar), 6.91 (d, *J* = 7.9 Hz, 1H, Ar), 6.81–6.89 (m, 4H, Ar), 5.59 (d, *J*_1,2_ = 3.7 Hz, 1H, H-1), 4.70 (dd, *J* = 14.7 Hz, 6.6 Hz, 1H, CH_2_N), 4.61 (dd, *J* = 14.7 Hz, 5.6 Hz, 1H, CH_2_N), 4.58 (dd, *J* = 14.7 Hz, 5.6 Hz, 1H, CH_2_N), 4.44 (dd, *J* = 14.7 Hz, 5.1 Hz, 1H, CH_2_N), 4.27 (m, 1H, H-6′), 4.21 (ddd, *J*_5,4_ = 10.1 Hz, *J*_5,6_ = 3.9 Hz, *J*_5,6_ = 1.6 Hz, 1H, H-5), 4.10–4.17 (m, 3H, H-5′, H-6, H-6′), 4.08 (d, *J*_3′,4′_ = 7.7 Hz, 1H, H-3′), 4.05 (dd, *J*_6,6_ = 10.2 Hz, *J*_6,5_ = 3.9 Hz, 1H, H-6), 3.90 (dd, *J*_4′,3′_ = 7.7 Hz, *J*_4′,5′_ = 7.7 Hz, 1H, H-4′), 3.64 (s, 3H, CH_3_), 3.58 (d, *J*_1′,1′_ = 11.0 Hz, 1H, H-1′), 3.52 (dd, *J*_3,2_ = 9.4 Hz, *J*_3,4_ = 9.2 Hz, 1H, H-3), 3.50 (s, 3H, CH_3_), 3.48 (s, 3H, CH_3_), 3.45 (s, 3H, CH_3_), 3.41 (s, 3H, CH_3_), 3.41 (d, *J*_1′,1′_ = 11.0 Hz, 1H, H-1′), 3.34 (s, 3H, CH_3_), 3.29 (dd, *J*_4,3_ = 9.2 Hz, *J*_4,5_ = 10.1 Hz, 1H, H-4), 3.19 (dd, *J*_2,1_ = 3.7 Hz, *J*_2,3_ = 9.4 Hz, 1H, H-2) ppm; ^13^C NMR: *δ* = 163.14 (C=O), 163.08 (C=O), 159.16, 158.93 (2 × C–Ar), 148.63, 148.54 (2 × C-pyridine), 139.25, 139.13 (2 × C–Ar), 139.13 (C-pyridine), 129.96, 129.92 (2 × C–Ar), 125.17, 125.14 (2 × C-pyridine), 121.11, 120.84, 114.41, 114.17, 113.74, 112.95 (6 × C–Ar), 104.07 (C-2′), 89.93 (C-1), 84.93 (C-3′), 83.61 (C-4′), 83.23 (C-3), 81.51 (C-2), 79.45 (C-4), 78.37 (C-5′), 74.19 (C-1′), 69.70 (C-5), 69.23 (C-6′), 66.77 (C-6), 60.69, 60.47, 59.34, 58.53, 58.41, 58.22 (6 × OCH_3_), 43.66, 43.63 (2 × CH_2_N) ppm; HRMS (ESI): calcd for C_39_H_49_N_3_O_13_Na [M + Na]^+^ 790.3158, found 790.3125.

#### *6,6′*-*Di*-*O*-*[[benzene*-*1,3*-*diyl*-*bis(carbonylaminomethyl)]*-*4,4′*-*diphenyl]*-*1′,2,3,3′,4,4′*-*hexa*-*O*-*methylsucrose* (**10c**, C_40_H_50_N_2_O_13_)

White solid; yield: 93 mg (71 %); m.p.: 144 °C; TLC: *R*_*f*_ = 0.35 (AcOEt); [*α*]_D_^24^ = +58.2° cm^2^ g^−1^; IR: $\overline{v}$ = 3,301, 3,064, 2,982, 2,931, 2,831, 1,649, 1,613, 1,586, 1,542, 1,514, 1,455, 1,422, 1,359, 1,319, 1,300, 1,248, 1,160, 1,101, 1,024, 983, 951, 824, 754, 700, 603, 580 cm^−1^; ^1^H NMR: *δ* = 7.88 (d, *J* = 7.8 Hz, 1H, isophthalic), 7.85 (d, *J* = 7.6 Hz, 1H, isophthalic), 7.56 (s, 1H, isophthalic), 7.45 (dd, *J* = 7.8 Hz, 7.6 Hz, 1H, isophthalic), 7.22 (d, *J* = 8.5 Hz, 2H, Ar), 7.07 (d, *J* = 8.5 Hz, 2H, Ar), 6.91 (d, *J* = 8.5 Hz, 2H, Ar), 6.78 (d, *J* = 8.5 Hz, 2H, Ar), 6.61 (br s, 1H, NH), 6.56 (br s, 1H, NH), 5.55 (d, *J*_1,2_ = 3.7 Hz, 1H, H-1), 4.45 (dd, *J* = 13.9 Hz, 5.2 Hz, 1H, CH_2_N), 4.39 (dd, *J* = 13.8 Hz, 6.7 Hz, 1H, CH_2_N), 4.35–4.39 (m, 2H, H-6′, CH_2_N), 4.32 (dd, *J* = 13.8 Hz, 4.8 Hz, 1H, CH_2_N), 4.28 (m, 1H, H-5), 4.20 (ddd, *J*_5′,4′_ = 8.3 Hz, *J*_5′,6′_ = 6.7 Hz, *J*_5′,6′_ = 3.3 Hz, 1H, H-5′), 4.14 (d, *J*_3′,4′_ = 7.9 Hz, 1H, H-3′), 4.13 (m, 1H, H-6), 4.09 (dd, *J*_6,6_ = 9.8 Hz, *J*_6,5_ = 5.3 Hz, 1H, H-6), 4.05 (dd, *J*_6′,6′_ = 9.9 Hz, *J*_6′,5′_ = 3.3 Hz, 1H, H-6′), 4.03 (dd, *J*_4′,3′_ = 7.9 Hz, *J*_4′,5′_ = 8.3 Hz, 1H, H-4′), 3.65 (s, 3H, CH_3_), 3.57 (d, *J*_1′,1′_ = 11.0 Hz, 1H, H-1′), 3.56 (s, 3H, CH_3_), 3.56 (m, 1H, H-3), 3.541 (s, 3H, CH_3_), 3.535 (s, 3H, CH_3_), 3.48 (s, 3H, CH_3_), 3.44 (s, 3H, CH_3_), 3.42 (d, *J*_1′,1′_ = 11.0 Hz, 1H, H-1′), 3.26 (dd, *J*_4,3_ = 9.2 Hz, *J*_4,5_ = 9.8 Hz, 1H, H-4), 3.18 (dd, *J*_2,1_ = 3.7 Hz, *J*_2,3_ = 9.6 Hz, 1H, H-2) ppm; ^13^C NMR: *δ* = 167.25 (C=O), 166.87 (C=O), 158.47, 158.42 (2 × C–Ar), 134.88, 134.88, 131.03, 130.99 (4 × C-isophthalic), 130.48, 129.80 (2 × C–Ar), 129.71 (C-isophthalic), 129.71 (2C-Ar), 128.59 (2C-Ar), 123.88 (C-isophthalic), 114.80 (2C-Ar), 114.71 (2C-Ar), 103.74 (C-2′), 88.89 (C-1), 84.64 (C-3′), 83.72 (C-4′), 83.28 (C-3), 81.68 (C-2), 79.84 (C-4), 78.89 (C-5′), 74.22 (C-1′), 69.77 (C-5), 69.44 (C-6′), 67.69 (C-6), 60.71, 60.50, 59.38, 58.82, 58.69, 58.44 (6 × OCH_3_), 43.72, 43.39 (2 × CH_2_N) ppm; HRMS (ESI): calcd for C_40_H_50_N_2_O_13_Na [M + Na]^+^ 789.3202, found 789.3203.

#### *6,6′*-*Di*-*O*-*[[pyridine*-*1,3*-*diyl*-*bis(carbonylaminomethyl)]*-*4,4′*-*diphenyl]*-*1′,2,3,3′,4,4′*-*hexa*-*O*-*methylsucrose* (**11c**, C_39_H_49_N_3_O_13_)

White solid; yield: 97 mg (74 %); m.p.: 115 °C; TLC: *R*_*f*_ = 0.37 (AcOEt); [*α*]_D_^21^ = +28.4° cm^2^ g^−1^; IR: $\overline{v}$ = 3,330, 2,982, 2,932, 2,831, 1,671, 1,613, 1,585, 1,535, 1,514, 1,449, 1,363, 1,301, 1,287, 1,248, 1,151, 1,100, 1,023, 1,003, 984, 949, 879, 827, 753, 677, 646, 603, 582 cm^−1^; ^1^H NMR: *δ* = 8.35 (dd, *J* = 7.8 Hz, 1.2 Hz, 1H, pyridine), 8.32 (dd, *J* = 7.8 Hz, 1.2 Hz, 1H, pyridine), 8.05 (t, *J* = 7.8 Hz, 1H, pyridine), 7.69 (dd, *J* = 4.6 Hz, 5.4 Hz, 1H, NH), 7.63 (dd, *J* = 4.6 Hz, 4.8 Hz, 1H, NH), 7.26 (d, *J* = 8.7 Hz, 2H, Ar), 7.02 (d, *J* = 8.7 Hz, 2H, Ar), 6.93 (d, *J* = 8.7 Hz, 2H, Ar), 6.73 (d, *J* = 8.7 Hz, 2H, Ar), 5.52 (d, *J*_1,2_ = 3.7 Hz, 1H, H-1), 4.67 (dd, *J* = 5.5 Hz, 14.2 Hz, 1H, CH_2_N), 4.57 (dd, *J* = 5.9 Hz, 14.9 Hz, 1H, CH_2_N), 4.53 (dd, *J*_6′,5′_ = 6.8 Hz, *J*_6′,6′_ = 10.0 Hz, 1H, H-6′), 4.46 (dd, *J* = 4.2 Hz, 14.9 Hz, 1H, CH_2_N), 4.42 (dd, *J* = 4.1 Hz, 14.2 Hz, 1H, CH_2_N), 4.34 (ddd, *J*_5,6_ = 1.3 Hz, *J*_5,6_ = 6.6 Hz, *J*_5,4_ = 10.3 Hz, 1H, H-5), 4.24 (ddd, *J*_5′,6′_ = 3.2 Hz, *J*_5′,6′_ = 6.8 Hz, *J*_5′,4′_ = 7.6 Hz, 1H, H-5′), 4.22 (dd, *J*_6,5_ = 1.3 Hz, *J*_6,6_ = 9.6 Hz, 1H, H-6), 4.15 (dd, *J*_4′,3′_ = 7.9 Hz, *J*_4′,5′_ = 7.6 Hz, 1H, H-4′), 4.12 (d, *J*_3′,4′_ = 7.9 Hz, 1H, H-3′), 4.10 (dd, *J*_6,5_ = 6.6 Hz, *J*_6,6_ = 9.6 Hz, 1H, H-6), 4.08 (dd, *J*_6′,6′_ = 10.0 Hz, *J*_5′,6′_ = 3.2 Hz, 1H, H-6′), 3.65 (s, 3H, CH_3_), 3.58 (s, 6H, CH_3_), 3.57 (d, *J*_1′,1′_ = 11.0 Hz, 1H, H-1′), 3.55 (m, 4H, H-3, CH_3_), 3.47 (s, 3H, CH_3_), 3.45 (s, 3H, CH_3_), 3.40 (d, *J*_1′,1′_ = 11.0 Hz, 1H, H-1′), 3.15 (dd, *J*_2,1_ = 3.7 Hz, *J*_2,3_ = 9.6 Hz, 1H, H-2), 3.13 (dd, *J*_4,3_ = 8.7 Hz, *J*_4,5_ = 10.3 Hz, 1H, H-4) ppm; ^13^C NMR: *δ* = 162.90 (C=O), 162.77 (C=O), 158.49, 158.42 (2 × C–Ar), 148.59, 148.43, 139.23 (3 × C-pyridine), 129.70 (2C-Ar), 129.61, 129.11 (2 × C–Ar), 128.17 (2C-Ar), 124.81, 124.76 (2 × C-pyridine), 114.92 (2C-Ar), 114.70 (2C-Ar), 103.71 (C-2′), 88.70 (C-1), 84.51 (C-4′), 83.70 (C-3′), 83.34 (C-3), 81.73 (C-2), 80.20 (C-4), 79.01 (C-5′), 73.99 (C-1′), 69.93 (C-5), 69.42 (C-6′), 68.06 (C-6), 60.68, 60.47, 59.34, 58.91, 58.75, 58.42 (6 × OCH_3_), 43.66, 43.06 (2 × CH_2_N) ppm; HRMS (ESI): calcd for C_39_H_49_N_3_O_13_Na [M + Na]^+^ 790.3158, found 790.3196.
